# Winery by-products as a feed source with functional properties: dose–response effect of grape pomace, grape seed meal, and grape seed extract on rumen microbial community and their fermentation activity in RUSITEC

**DOI:** 10.1186/s40104-023-00892-7

**Published:** 2023-07-10

**Authors:** Ratchaneewan Khiaosa-ard, Mubarik Mahmood, Elsayed Mickdam, Cátia Pacífico, Julia Meixner, Laura-Sophie Traintinger

**Affiliations:** 1 Institute of Animal Nutrition and Functional Plant Compounds, Department for Farm Animals and Veterinary Public Health, University of Veterinary Medicine Vienna, Veterinaerplatz 1, 1210 Vienna, Austria; 2 Animal Nutrition Section, Department of Animal Sciences, Sub Campus Jhang, University of Veterinary and Animal Sciences, 12 Km Chiniot Road, Jhang 35200, Lahore, Pakistan; 3 Nutrition and Clinical Nutrition Department, Faculty of Veterinary Medicine, South Valley University, Qena, 83523 Egypt; 4 Present address: Biome Diagnostics GmbH, Vienna, Austria

**Keywords:** Functional feed, Grape pomace, Grape seed, Protein degradation, Rumen microbiota

## Abstract

**Background:**

Grape and winery by-products have nutritional values for cattle and also contain functional compounds like phenols, which not only bind to protein but can also directly affect microbiota and their function in the rumen. We characterized the nutritional and functional effects of grape seed meal and grape pomace as well as an effective dosage of grape phenols on ruminal microbiota and fermentation characteristics using a rumen simulation technique.

**Results:**

Six diets (each *n* = 8) were compared including a control diet (CON, no by-product), a positive control diet (EXT, CON + 3.7% grape seed extract on a dry matter (DM) basis), two diets with grape seed meal at 5% (GS-low) and 10% (GS-high), and two diets with grape pomace: at 10% (GP-low) and 20% (GP-high), on a DM basis. The inclusion of the by-product supplied total phenols at 3.4%, 0.7%, 1.4%, 1.3%, and 2.7% of diet DM for EXT, GS-low, GS-high, GP-low, and GP-high, respectively. Diets were tested in four experimental runs. All treatments decreased ammonia concentrations and the disappearances of DM and OM (*P* < 0.05) compared to CON. EXT and GP-high lowered butyrate and odd- and branch-chain short-chain fatty acids while increased acetate compared to CON (*P* < 0.05). Treatments did not affect methane formation. EXT decreased the abundance of many bacterial genera including those belonging to the core microbiota. GP-high and EXT consistently decreased *Olsenella* and *Anaerotipes* while increased *Ruminobacter* abundances.

**Conclusion:**

The data suggest that the inclusion of winery by-products or grape seed extract could be an option for reducing excessive ammonia production. Exposure to grape phenols at a high dosage in an extract form can alter the rumen microbial community. This, however, does not necessarily alter the effect of grape phenols on the microbial community function compared to feeding high levels of winery by-products. This suggests the dominant role of dosage over the form or source of the grape phenols in affecting ruminal microbial activity. In conclusion, supplementing grape phenols at about 3% of diet DM is an effective dosage tolerable to ruminal microbiota.

**Supplementary Information:**

The online version contains supplementary material available at 10.1186/s40104-023-00892-7.

## Background

Phenol compounds elicit various effects such as protein binding, shifting ruminal microbial composition, and modulating microbial activity, though the pattern and intensity differ depending on the dosage and botanical origin of the phenolic compounds [1]. Research thus far has focused on extracts and bioactive compounds of plants, many of which are from exotic and intensively managed sources such as extracts of tree bark, leaves, and purified plant bioactive compounds [1], while agro-industrial by-products from phenol-rich plants have been underexplored. Therefore, it seems reasonable to harness benefits from these more common resources especially those with high abundances such as grape and winery by-products.

Grapes from the common grapevine (*Vitis vinifera* L.) are grown worldwide and are common food sources of phenolic compounds, particularly flavonoids [2]. Health benefits of grape products such as wine and grape seed extracts have been previously described [3]. Production of wine, grape seed oil, and grape-derived supplements results in considerable amounts of by-products like grape pomace and grape seed meal. Turning grape and winery by-products into ruminant feed sources is an effective way to upcycle these, otherwise, problematic wastes due to the adverse effects of tannins on soil quality and the ecosystem [4]. Grape and winery by-products may offer a twofold benefit as a potential feed source and a source of functional compounds. Despite the great availability of these resources, nutrition research in feeding grape and winery by-products to ruminants is small. Some data are available in small ruminant species but less so in cattle and the existing data are inconclusive due to large variations in feeding conditions and dosages [5]. Research so far has suggested a mitigating effect of grape and winery by-products [6, 7] on methane production, in addition to increasing milk protein yield [2], which indicates that grape phenolics may influence ruminal N metabolism, thus sparing dietary protein losses to ruminal degradation. However, little is known about how ruminal microbiota respond to grape phenols and thus the effective dose and tolerable level are not known. It is important to note that the effects of phytoconstituents on ruminal microbiota and fermentation also depend on the diet in addition to the supplementation dosage [8]. In the present research, we studied the effects of different inclusion levels of grape seed meal and grape pomace in comparison to grape seed extract supplementation using a rumen simulation technique (RUSITEC). In vitro models such as continuous cultures are valuable tools for nutrition and microbiological research in ruminants, which help to reduce the need for animals and thus the ethical issues related. The grape and winery by-products were included in diets with high-quality hay, aiming to counteract the drawback of high-quality hay in producing excessive ammonia concentrations shown before [9]. High-quality hay contains high protein and highly digestible carbohydrates [9], while grape seed meal and grape pomace are low in protein but rich in fibrous carbohydrates [5, 10, 11]. Therefore, besides the functional effects of the phenolic compounds, effects of nutrient shifts from the inclusion of these by-products on ruminal fermentation and microbial community can also be anticipated. Ultimately, our goals were (1) to distinguish the nutritional and functional effects of grape seed meal and grape pomace and (2) to ascertain an effective dosage of grape phenols tolerable to ruminal microorganisms. To reach these goals, by-product treatments were tested against both positive control (grape seed extract addition) and negative control.

## Methods

### Dietary treatments

Six dietary treatments were investigated in the present study. All diets were high-forage diets containing high-quality hay and a grain mix, and the content thereof varied depending on the substitution from grape seed meal or grape pomace (Table 1). The control diet (CON) contained, on a DM basis, 70% high-quality hay and 30% grain mix. The second diet was EXT, which was the CON diet supplemented with a commercial grape seed extract (Nature Love® OPC Grape Seed Extract, Tauron Ventures GmbH, Düsseldorf, Germany) at 3.7% of the CON diet DM. The remaining four treatments were two inclusion levels of grape pomace at 10% (GP-low) and 20% (GP-high) and two levels of grape seed meal at 5% (GS-low) and 10% (GS-high) of diet DM. The by-products substituted both hay and grain mix in the diet intending to keep similar nutrient chemical compositions and forage to concentrate ratios of all diets as much as possible (Table 1). The high-quality hay consisted mainly of a mixture of first- and second-cut *Lolium perenne* harvested at the beginning of ear emergence followed by indoor drying [12]. The grain mix consisted of a mixture of starchy cereal grains (barley, wheat, and corn) and a commercial vitamin and supplement (more detail see Table 1). The grape pomace and grape seed meal came from red wine production by a local producer in Gumpoldskirchen, Austria. The antioxidant capacity of the test grape pomace, grape seed meal, and grape seed extract was 10.3, 11.3, and 121.4 mmol of Trolox/g DM, respectively. The inclusion levels of high-quality hay were adapted to the range formerly tested in early lactation cows [13]. The levels of winery by-products were based on previous test dosages and recommendations [6, 7]. All ingredients were ground with a Wiley mill (Pulverisette 25/19, FRITSCH GmbH, Idar-Oberstein, Germany) to pass through a 6-mm sieve before use.

**Table 1:** Ingredients, chemical composition, and total phenol contents of treatments without (CON) or with different inclusion levels of grape pomace (GP), grape seed meal (GS) or supplemented with a grape seed extract (EXT)

Item	CON	EXT	GP-low	GP-high	GS-low	GS-high
Ingredient composition, % of DM (otherwise stated)						
Hay^a^	70	70	65	56	70	65
Grain mix^b^	30	30	25	24	25	25
Grape pomace	0	0	10	20	0	0
Grape seed meal	0	0	0	0	5	10
Grape seed extract, % of basal diet DM	0	3.7	0	0	0	0
Chemical composition, % of DM (otherwise stated)						
Dry matter, % of fresh matter	89.21	89.21	89.54	90.81	89.52	89.78
Organic matter	92.29	92.29	92.57	92.54	92.61	92.92
Crude protein	18.70	18.70	19.13	18.30	19.82	18.84
Ether extract	2.58	2.58	3.66	3.39	2.48	2.55
Neutral detergent fiber	49.26	49.26	48.31	52.56	50.08	51.67
Acid detergent fiber	20.88	20.88	24.68	28.46	23.07	24.16
Non-fiber carbohydrates	21.75	21.75	21.47	18.29	20.23	20.26
Net energy lactation, MJ/kg DM	7.04	7.04	6.92	6.83	6.83	6.66
Total phenol content^c^						
Daily supply, % of diet DM	2.9	6.4	4.0	5.0	3.6	4.1
Thereof grape by-product, % of diet DM	0	3.4	1.3	2.7	0.7	1.4

^a^Contained (on dry matter basis), 6.84 MJ/kg net energy lactation, 91.7% organic matter, 21.9% crude protein; 20.5% water soluble carbohydrates, 45.2% neutral detergent fiber and 2.2% ether extract

^b^Contained (on dry matter basis, %) 21.6 barley; 21.6 wheat; 51.7 corn; and 5.2 vitamin and mineral supplement (Rindavit TMR 11 ASS-CO + ATG; H. Wilhelm Schaumann GmbH & Co KG, Brunn/Gebirge, Austria)

^c^Expressed as catechin equivalents

### RUSITEC procedure

The experiment was performed using two RUSITEC systems, each system consisting of six bioreactors. There were four experimental runs. In each run, all six treatments were tested in duplicate (total *n* = 8 per dietary treatment). Each experimental run included a 5-day adaptation period followed by a 5-day sampling period. The RUSITEC procedure was performed following a standard procedure described previously [14]. In short, the inoculum (liquid and solids) used in each experimental run was obtained from two rumen-cannulated non-pregnant dry cows fed hay ab libitum with a daily allowance of 0.5 kg of commercial concentrates (KuhKorn PLUS Energie, Garant-Tiernahrung GmbH, Pölchlarn, Austria). Thus, the forage to concentrate ratios of the donor cows’ diet and experimental diets differed. The liquid contents of both donor cows were strained through a four-layer of medical gauze (1 mm pore size) and pooled forming one uniform batch of inoculum. The pH and redox potential of the inoculum were 6.69 ± 0.40 and −304 ± 33 mV, respectively (mean ± SD). Concentrations of fermentation acids in the ruminal fluid of each donor cow are presented in Supplementary Table 1. Each bioreactor unit was inoculated with 600 mL of the strained ruminal fluid and 100 mL of the pre-warmed McDougal’s buffer [15]. On the first day of the experimental run, two nylon bags (140 mm × 70 mm, 150 µm pore size, Fa. Linker Industrie-Technik GmbH, Kassel, Germany) were added to each bioreactor, with one bag containing mixed solid contents, and another bag containing 12 g DM of the respective diet. Subsequently, an anaerobic condition of the system was established via flushing with a stream of N_2_. All bioreactors were kept at 39.5 °C regulated by thermostatically controlled water baths. Bioreactors were continuously infused with the buffer via a 12-channel peristaltic pump (model ISM932, Ismatec, Idex Health & Science GmbH, Wertheim, Germany) at a rate of 375 mL/d (2.5% of fermentation volume per h) throughout the experiment. Feedbag exchanges were done daily. After the first 24 h, the bag with solid digesta was replaced with a new bag containing the respective diet and on the subsequent days, the spent bag was then replaced by a new bag, with each bag being subjected to 48 h of incubation. The fermentation gas was collected in a gas-tight aluminum bag (TECOBAG 8 L, Tesseraux Spezialverpackungen, Bürstadt, Germany) which was exchanged with an emptied bag daily. The anaerobic conditions were re-established immediately after the feedbag exchange of each bioreactor.

### Sampling and chemical analyses

Sampling was performed after the 5-day adaptation allowing system equilibrium and microbial adaption to the respective test diet. About 5 mL of the fermentation liquid was used for measurement of pH and redox using a pH meter (Seven Multi TM, Mettler-Toledo GmbH, Schwerzenbach, Switzerland) equipped with separate electrodes (InLab Expert Pro-ISM for pH and Pt4805-DPA-SC-S8/120 for redox; Mettler-Toledo GmbH, Schwerzenbach, Switzerland). An aliquot of fermentation liquid was collected and stored at −20 °C until the analysis of volatile fatty acids (VFA) using gas chromatography [16] with small modifications and ammonia using the indophenol reaction method [17]. The daily volume of fermentation gases was measured by water displacement [18]. The gas composition was measured using an infrared detector (ATEX Biogas monitor Check BM 2000, Ansyco, Karlsruhe, Germany). The 48-h incubated feedbags were machine-washed (cold wash and gentle cycles) for 30 min and the excessive water was squeezed out before being stored at −20 °C until analysis. The original feedstuffs and 48-h incubated feed residues were analyzed for the contents of DM, ash, CP, acid detergent fiber (ADF), neutral detergent fiber (NDF), and ether extract (EE) following the procedures of VDLUFA [19]. The contents of organic matter (OM: DM − ash) and non-fiber carbohydrates (100 − ash − CP − NDF − EE) were calculated. The nutrient degradation was estimated from the differences between the supplied and that recovered amounts. In addition, the production of ammonia and methane was normalized by the unit of relevant substrate degraded to determine the fermentation efficiency.

Diet ingredients and grape seed extract were analyzed for the total phenol content (free and bound forms). About 400 mg of each material was mixed with 18 mL of 60% ethanol and 2.5 mL of 2 mol/L HCl. The acid hydrolysis was carried out at 95° C for 2 h. After cooling, the volume was made up to 25 mL with 60% ethanol and was then filtrated through cellulose paper. Concentrations of phenolics in the extract were determined by the Folin-Ciocalteu method using a Folin-Ciocalteu reagent and sodium carbonate solution in which the colorimetric reactions were adapted to be measured with a microplate absorbance reader (iMark, Bio Rad Laboratories, Inc., Hercules, CA, USA). After 1 h of resting in the dark, the absorbance was measured at 750 nm. The phenolics were expressed as catechin equivalents.

The antioxidant capacity of the grape seed meal, grape pomace, grape seed extract, and d-10 fermentation liquid was measured using a ferric-reducing antioxidant power (FRAP) assay adapted from the method described previously [20]. This test relies on the ability of antioxidants to reduce ferric (Fe^3^^+^) ions. The winery by-products were subjected to an ethanol extraction and the extract was used for the analysis. Shortly, 24 µL of each sample were tested into a 96-well microplate followed by the addition of 180 µL of a pre-warmed (37 °C) working reagent (25 mL of acetic acid buffer, 2.5 mL of a 2,4,6-tripyridl-s-triazin solution and 2.5 mL of FeCl_3_·6H_2_O). After 5 min of reaction, the absorbance was measured at 490 nm using a thermostat microplate spectrophotometer (xMark™, Bio-Rad Laboratories, Hercules, CA, USA). Measurements were done in duplicates and quantifications were done with a set of standards and blank. The quantification was expressed as Trolox equivalents.

### DNA extraction, amplification, and sequencing

Microbiota samples were obtained from d-10 fermentation liquid samples from three experimental runs (*n* = 6 per treatment). DNA isolation and purification were performed using the DNeasy PowerSoil Pro Kit (Qiagen, Hilden, Germany) with minor modifications [14]. Briefly, 800 μL of fermenter fluid was added to bead-beating tubes and mixed with solution C1, followed by incubation at 95 °C for 5 min. After centrifugation at 10,000 × *g* for 2 min, the supernatant was recovered and placed on ice. The pellet was mixed with 100 µL of 100 mg/mL lysozyme and 10 µL of 2.5 U/mL mutanolysin and kept at 37 °C for 30 min. Afterward, 21.3 µL of 18.8 mg/mL proteinase K was added and the mixture was incubated at 37 °C for 1 h. Pellets homogenization was obtained through bead beating (FastPrep-24, MP Biomedical, Santa Ana, CA, USA) and the supernatant was collected after centrifugation. Sequential centrifugation steps allowed the removal of cell debris and PCR inhibitors. The supernatant was transferred to new tubes and DNA was eluted in 100 µL of C6 buffer. Measurement of total DNA was performed using the Qubit Fluorometer 2.0 (Qubit dsDNA HS Assay Kit, Thermo Fisher Scientific, Vienna, Austria) according to the manufacturer’s instructions. 16S rRNA sequencing was performed using Illumina MiSeq paired-ends technology (Microsynth AG, Balgach, Switzerland). Targeted amplification of the hypervariable region V4 of bacterial 16S rRNA gene (2 × 250 bp) was performed using the primers 515F (5′-GTGCCAGCMGCCGCGGTAA-3′) and 806R (5′-GGACTACHVGGGTWTCTAAT-3′). Multiplexed libraries were constructed by ligating sequencing adapters and indices onto purified PCR products using the Nextera XT Sample Preparation Kit (Illumina, Balgach, Switzerland). After trimming adapters and primers, corresponding overlapping paired-end reads were stitched by Microsynth AG, Balgach, Switzerland.

### Bioinformatics and data analysis

Merged reads were processed using the software package Quantitative Insights into Microbial Ecology (QIIME2 v2020.2) [21]. Preliminary read quality was assessed using FASTQC v. 0.11.5 [22]. Sequences were further quality filtered using the q-score-joined plugin with a minimum acceptable PHRED score of 20 (–p-min-quality 20). Prior to denoising into amplicon sequence variants (ASVs) with Deblur [23], all reads were trimmed to a length of 250 nucleotides and features with an abundance below 10 were removed from the analysis. Representative sequences and feature table were filtered to exclude all features classified as mitochondria or chloroplast sequences. The resulting features were aligned with mafft [24] and used to construct a phylogenetic tree with fasttree2 [25]. Taxonomy was assigned to ASVs using a classify-sklearn naïve Bayes taxonomy classifier trained on the 515F/806R primer set against the SILVA 132 99% OTUs reference sequences (https://www.arb-silva.de, version 132). The rooted tree, taxonomy file, and filtered feature table were used as input to the R package phyloseq [26] in RStudio (RStudio Team, 2020, Boston, MA, USA). Prediction of functional composition was performed using PICRUSt2 [27]. Raw read sequences have been submitted to the National Center for Biotechnology Information (NCBI) Sequence Read Archive (SRA) database under the accession number PRJNA817202.

### Statistical analysis

To match with nutrient degradation data, daily ruminal fermentation characteristic variables were averaged across the sampling days. The averaged data and nutrient degradation data were then analyzed using the MIXED procedure in SAS (version 9.4, SAS Institute Inc., Cary, NC, USA). The mixed model consisted of the fixed factor of dietary treatment and the random effect of the experimental run and bioreactor. Alpha-diversity indices (number of observed ASVs, Fisher, Shannon, and Simpson indices) were calculated in phyloseq. All diversity indices had a normal distribution according to the Shapiro–Wilk test (*P* > 0.05). Differences in community richness and diversity were analyzed with the MIXED procedure of SAS (version 9.4, SAS Institute Inc., Cary, NC, USA) using the same statistical model as given above. Analysis of similarity (ANOSIM) for multivariate data was calculated using Aitchison, weighted and unweighted UniFrac, and Bray–Curtis distance metrics. The analysis was carried out within phyloseq, using the vegan package. Comparisons of the microbial abundances among treatments at family and genus levels were performed using MaAsLin2 [28] using default settings. The false discovery rate [29] corrected *P*-values ≤ 0.05 were considered to indicate significant differences. Spearman correlations were calculated using Hmisc v 4.6.0. For network analysis, target pathways are related to N metabolism and VFA focusing on acetate and butyrate according to MetaCyc [30]. The results of the mixed models are reported as least squares (LS) means ± standard error of the mean (SEM). When the treatment effect was significant (*P* < 0.05), pairwise comparisons of GP, GS, and EXT treatments against that of CON were performed using Dunnett’s test. In addition, a contrast analysis of GP or GS vs. EXT and GP vs. GS was performed. Significance was declared at *P* ≤ 0.05 and a trend was set at 0.05 < *P* ≤ 0.10.

## Results

### Ruminal fermentation characteristics

As shown in Table 2, the pH of the fermentation liquid was affected by treatment (*P* = 0.006) as GP-low and GS-high raised the pH compared to CON and EXT (*P* < 0.05), while the redox potential remained unaffected by treatment. The inclusion of grape and winery by-products decreased ammonia concentration (*P* < 0.001). The suppression was strongest with GP-high with a 22% reduction compared to that of CON, followed by EXT (17% reduction), GS-high and GP-low (10%–12% reduction), and GS-low (5.6% reduction) (*P* < 0.05). The contrast analysis also showed an effect of the type of winery by-product on ammonia concentration (GP vs. GS, *P* < 0.001). No difference between by-product treatments and CON was detected for the fermentation gas volume and gas composition. The production of fermentation gas was similar among treatments. EXT lowered methane percentage when compared with the by-product treatments (*P* < 0.01).

**Table 2:** Fermentation characteristics of fermentation liquid as affected by dietary treatment^a^

Item	Treatment						SEM	Overall *P-*value	Contrast *P*-value		
	CON	EXT	GP-low	GP-High	GS-low	GS-high			EXT vs. GP	EXT vs. GS	GP vs. GS
pH	6.57	6.58	6.64^*^	6.62	6.61	6.65^*^	0.04	0.006	0.021	0.024	0.945
Redox, mV	−243.8	−239.0	−241.7	−232.2	−246.4	−231.9	6.7	0.276	0.749	0.979	0.670
Ammonia, mmol/L	14.45	12.00^*^	12.93^*^	11.26^*^	13.64^*^	12.70^*^	0.24	< 0.001	0.676	< 0.001	< 0.001
Methane, %	9.90	9.55	10.53	10.05	10.49	10.37	0.32	0.011	0.005	0.001	0.489
Carbon dioxide, %	84.33	84.19	83.56	83.83	83.23^*^	83.62	0.48	0.032	0.097	0.014	0.489
Methane, mL/d	87	78	86	79	84	81	7.80	0.250	0.305	0.262	0.892
Carbon dioxide, mL/d	734	685	677^+^	651^*^	667	648^*^	44.76	0.094	0.417	0.304	0.778
Total fermentation gas, mL/d	871	815	810	777^+^	801	775^*^	55.83	0.124	0.498	0.403	0.836
VFA, mmol/L	128.5	124.8	119.5^*^	120.4^*^	124.0	117.7^*^	5.6	< 0.001	0.016	0.044	0.584
VFA composition, %											
Acetate	48.57	50.35^*^	49.24	50.68^*^	48.89	49.53	0.73	< 0.001	0.338	0.009	0.031
Propionate	24.60	26.70	25.05	26.30	25.87	24.53	1.12	0.158	0.239	0.088	0.493
Butyrate	10.82	9.74^*^	10.76	9.62^*^	10.67	10.37	0.18	< 0.001	0.035	< 0.001	0.053
Isobutyrate	0.96	0.77^*^	0.92	0.87^*^	0.94	0.94	0.04	< 0.001	< 0.001	< 0.001	0.001
Valerate	8.09	6.76^*^	7.59	6.88^*^	7.26	7.86	0.43	0.014	0.181	0.030	0.267
Isovalerate	2.94	2.73	2.67^+^	2.56^*^	2.79	2.92	0.11	0.004	0.174	0.161	0.002
Caproate	3.08	2.31^*^	2.92	2.34^*^	2.75	2.89	0.29	0.008	0.102	0.012	0.233
Heptonate	0.96	0.68^*^	0.84	0.76^+^	0.82	0.92	0.11	0.013	0.072	0.008	0.228
Antioxidant capacity, µg/mL^b^	2.58	2.87	2.24	2.41	2.48	2.53	0.16	0.129	0.007	0.057	0.251

Values in the same row with * significantly differ from CON (*P* < 0.05) or with + tend to differ from CON (0.05 < *P* ≤ 0.10) according to Dunnett’s test

^a^Diets without (CON) or with different inclusion levels of grape pomace (GP-low and GP-high) or grape seed meal (GS-low and GS-high) or supplemented with grape seed extract (EXT)

^b^Expressed as Trolox equivalents

Both GP and GS-high treatments decreased VFA concentrations (5%–8% reduction) as compared to CON (*P* < 0.05) but only EXT and GP-high affected the VFA composition (Table 2). EXT and GP-high increased acetate percentage but decreased percentages of butyrate, isobutyrate, valerate, and caproate compared to CON (*P* < 0.05). Including grape pomace decreased the percentage of isovalerate compared to the grape seed meal inclusion (contrast analysis *P* = 0.002). The antioxidant capacity of fermentation liquid was unaffected by treatment.

All treatments except GS-low decreased the ruminal disappearance of DM and OM as compared to CON (*P* < 0.05, Table 3). The disappearance of CP was lower in EXT (−3.6%, *P* < 0.05) and GP-high (−3%, *P* < 0.10) than in CON. All treatments showed approx. 14%–18% reduction in ammonia concentration normalized with the amount of degraded CP compared to CON (*P* < 0.05). GP and GS treatments decreased the ash disappearance compared to CON and EXT. GP-low increased the EE disappearance (+ 16%, *P* < 0.05) but EXT tended to decrease the disappearance (−13%, *P* < 0.10) compared to CON. GP treatments lowered NDF disappearance compared to the other groups (*P* < 0.01). The disappearance of ADF was unaffected by treatment. As revealed by the contrast analysis, the inclusion of grape pomace decreased the degradation of DM, OM, CP, and NDF and increased EE degradation compared to the grape seed meal inclusion (*P* < 0.01). Fermentation efficiency as indicated by VFA concentration per unit of degraded OM was similar among all treatments, except for GP-low showing a lower value compared to CON (*P* < 0.10). Methane production per unit of degraded OM was not affected by treatment. When normalized by the unit of CP degraded, all by-product and EXT treatments performed similarly in decreasing ammonia concentrations compared to CON (*P* < 0.05).

**Table 3:** Nutrient degradation and fermentation efficiency as affected by dietary treatment^a^

Item	Treatment						SEM	Overall *P-*value	Contrast *P*-value		
	CON	EXT	GP-low	GP-High	GS-low	GS-high			EXT vs. GP	EXT vs. GS	GP vs. GS
Nutrient degradation, %											
Dry matter	65.03	62.49^*^	62.61^*^	61.32^*^	63.95^+^	62.78^*^	0.66	< 0.001	0.190	0.032	< 0.001
Organic matter	63.08	60.33^*^	60.65^*^	59.27^*^	62.04	60.91^*^	0.72	< 0.001	0.380	0.009	< 0.001
Crude protein	69.43	66.93^*^	69.34	67.19^+^	71.24	70.75	0.99	< 0.001	0.107	< 0.001	< 0.001
Ether extract	45.58	39.52^+^	52.82^*^	50.99	41.03	43.12	3.21	< 0.001	< 0.001	0.221	< 0.001
Neutral detergent fiber	47.24	46.24	39.74^*^	42.29^*^	46.51^+^	45.11	2.50	< 0.001	< 0.001	0.558	< 0.001
Acid detergent fiber	36.88	33.70	32.44	34.48	35.89	34.79	1.31	0.157	0.995	0.210	0.219
Fermentation efficiency											
Short-chain fatty acids, mmol/g degraded organic matter	9.60	9.41	8.87^+^	9.55	9.29	9.04	0.28	0.042	0.356	0.266	0.811
Methane, mL/g degraded organic matter	12.33	11.22	12.59	11.70	12.10	11.78	1.21	0.243	0.077	0.170	0.623
Ammonia, mmol/g degraded crude protein/d	4.88	4.08^*^	4.10^*^	4.01^*^	4.19^*^	4.15^*^	0.28	< 0.001	0.838	0.422	0.221

Values in the same row with * significantly differ from CON (*P* < 0.05) or with + tend to differ from CON (0.05 < *P* ≤ 0.10) according to Dunnett’s test

^a^Diets without (CON) or with different inclusion levels of grape pomace (GP-low and GP-high) or grape seed meal (GS-low and GS-high) or supplemented with grape seed extract (EXT)

### Ruminal microbiota

**Fig. 1:**
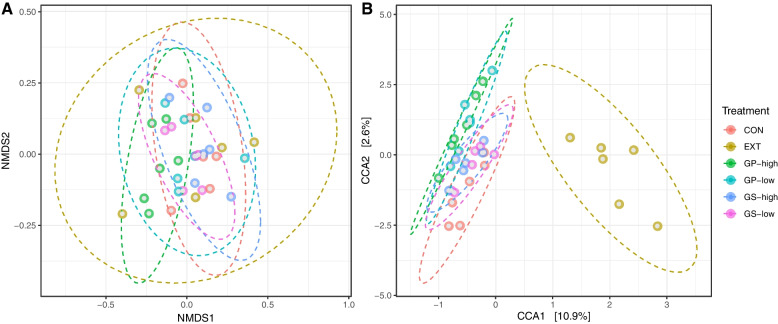
Beta-diversity determined using **A**: non-metric multidimensional scaling (NMDS) and **B**: canonical correspondence analysis (CCA). Treatments include diets without (CON) or with low and high inclusion levels of grape pomace (GP) or grape seed meal (GS) or supplemented with grape seed extract (EXT). Only CCA is affected by treatment (*P* = 0.046)

A total of 1,751,485 merged reads were imported into the analysis. From these, 1,749,120 high-quality reads were used for the downstream analysis of samples. The final dataset consisted of 4,322 ASVs and an average of 30,354 reads per sample. As shown in Table 4, an effect of the treatment was found for the number of observed ASVs (*P* = 0.01), particularly between CON and GP-high (*P* < 0.05). Alpha diversity indices (Shannon, Simpson, and Fisher) were similar among treatments. Treatments did not show different beta diversity as determined using non-metric multidimensional scaling but canonical correspondence analysis revealed a separation of EXT from the other treatments (*P* = 0.046, Fig. 1).

**Table 4:** Alpha-diversity of microbiota detected in fermentation liquid as affected by dietary treatment^a^

Item	Treatment						SEM	Overall *P-*value	Contrast *P-*value		
	CON	EXT	GP-low	GP-high	GS-low	GS-high			EXT vs. GP	EXT vs. GS	GP vs. GS
Number of observed ASVs^b^	929	811	883	660^*^	801	983	69.0	0.014	0.577	0.283	0.049
Shannon	4.79	4.69	4.67	4.72	4.63	4.79	0.14	0.821	0.966	0.875	0.889
Simpson	0.97	0.96	0.96	0.97	0.96	0.96	0.00	0.678	0.904	0.691	0.527
Fisher’s alpha	175	152^+^	168	143	159	181	11.0	0.056	0.762	0.121	0.110

Values in the same row with * significantly differ from CON (*P* < 0.05) or with + tend to differ from CON (0.05 < *P* ≤ 0.10) according to Dunnett’s test

^a^Diets without (CON) or with different inclusion levels of grape pomace (GP-low and GP-high) or grape seed meal (GS-low and GS-high) or supplemented with grape seed extract (EXT)

^b^Amplicon sequence variants

**Fig. 2:**
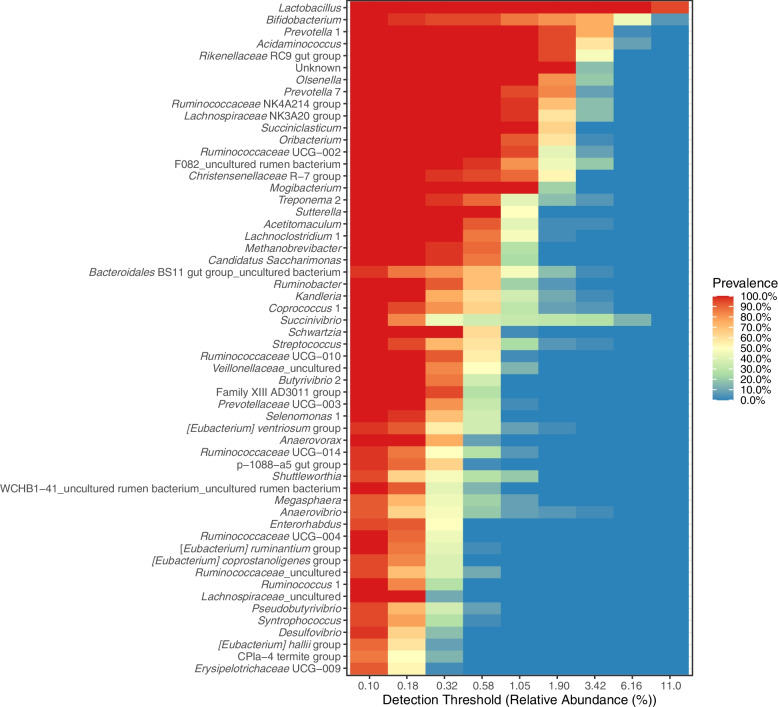
Shared and core microbiota of fermentation liquid samples

**Fig. 3:**
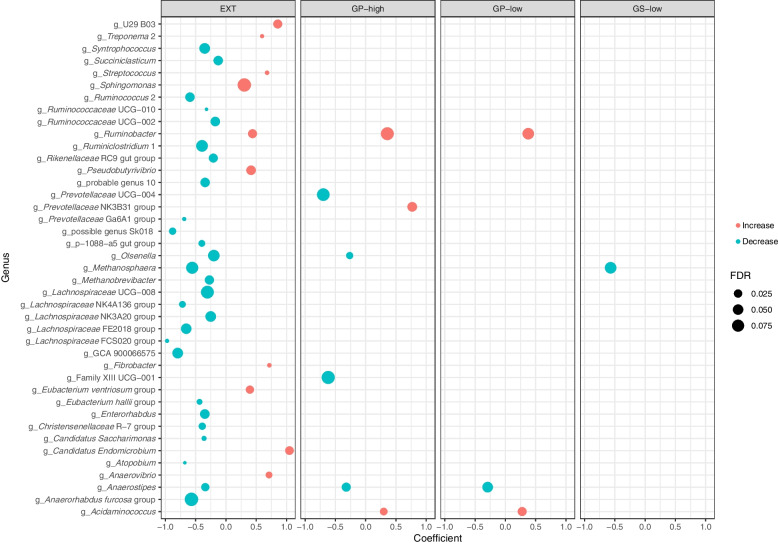
Treatments induced variation of the microbiota. Treatments include diets with low and high inclusion levels grape pomace (GP) or grape seed meal (GS) or supplemented with grape seed extract (EXT). Indicated changes (increase or decrease) are in comparison to the control diet without any winery by-product. GS-high shows no effect

Core microbiota at the genus level is depicted in Fig. 2. The top five classified genera were *Lactobacillus*, *Bifidobacterium, Prevotella, Acidaminococcus,* and *Rikenellaceae* RC9 gut group showing high relative abundances in most of the samples (at a prevalence of 80%–100%). High heterogeneity was observed for some genera. For instance, about half of the samples showed a relative abundance of *Succinivibrio* around 0.3%, and the rest of the samples showed higher abundances. Not all core genera were affected by treatment (Fig. 3). The top five core genera remained unaffected, while other main genera (with relative abundances of about 1% in most of the samples) classified as *Lachnospiraceae* NK3A20 group, *Succiniclasticum, Ruminococcaceae* UGC-002, *Chistensenellaceae* R-7 group were decreased and *Treponema* 2 was increased by EXT (*P* < 0.05). A tendency of the EXT effect was found for *Olsenella* (*P* < 0.075)*.* Less abundant genera were often decreased by EXT except for U29 BO3, *Streptococcus*, *Sphingomonas, Ruminobacter, Pseudobutyrivibrio, Fibrobacter, Eubacterium ventriosum* group, *Candidatus Endomicrobium,* and *Anaerovibrio*. Fewer genera were affected by GP and GS treatments. GP-low and GP-high decreased *Anaerostipes* but increased *Ruminobacter* (*P* < 0.05)*.* Both GP treatments decreased *Acidaminococcus* (*P* < 0.05)*.* Only GP-high decreased *Olsenella* and increased *Prevotellaceae* NK3B31 group (*P* < 0.05) and tended to decrease Family XIII UCG-001, and *Prevotellaceae* UGC-004 and(*P* < 0.010). GS-low decreased *Methanosphaera* (*P* = 0.05). There was no effect of GS-high on relative abundances at the genus level. *Methanobrevibacter* was the archaeal genus characterized as part of the core microbiota in the samples (Fig. 2). EXT decreased abundances of *Methanobrevibacter* and *Methanospahaera* (*P* ≤ 0.05) (Fig. 3).

**Fig. 4:**
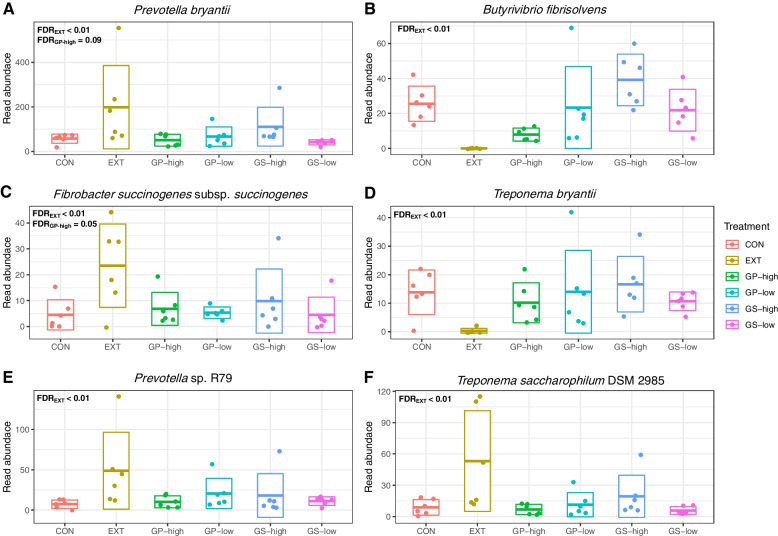
Key bacterial species affected by treatments including diets without (CON) or with low and high inclusion levels grape pomace (GP) or grape seed meal (GS) or supplemented with grape seed extract (EXT). FDR is a *P* value corrected for the false discovery rate

At the species level, EXT showed the strongest effect compared to the other treatments (Fig. 4). Compared to CON, EXT increased read abundances of *Prevotella bryantii, Fibrobacter succinogenes* subsp. *succinogenes*, *Prevotella* sp. R79 and *Treponema saccharophilum* DSM 2985 but decreased read abundances of *Butyrivibrio fibrisolvens* and *Treponema bryantii* (*P* < 0.01). GP-high tended to increase *Prevotella bryantii* (*P* = 0.09) and *Fibrobacter succinogenes* subsp. *succinogenes* (*P* = 0.05) compared to CON.

**Fig. 5:**
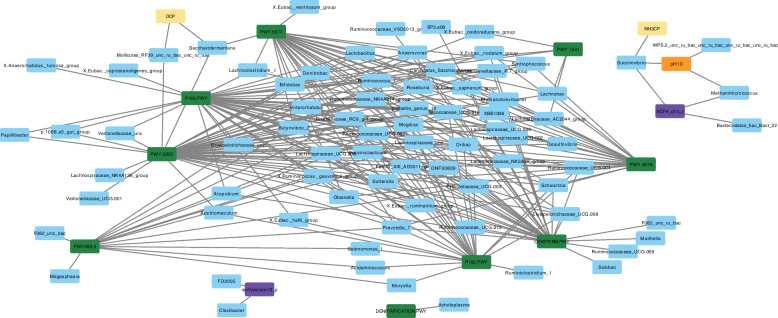
Network analysis of microbial genera showing positive or negative correlation coefficients above the threshold (0.70) with fermentation variables (DCP: crude protein degradation (%), NH3CP: ammonia concentration (mmol per g crude protein degraded), isoValerate10_p: d-10 isovalerate (%), and pH10: d 10-pH) or targeted pathways (CENTFERM-PWY: pyruvate fermentation to butanoate, P162-PWY: *L*-glutamate degradation V (via hydroxyglutarate), P163-PWY: *L*-lysine fermentation to acetate and butanoate, PWY-5022: 4-aminobutanoate degradation V, PWY-5676: Acetyl-CoA fermentation to butanoate II, PWY-5677: Succinate fermentation to butanoate, DENITRIFICATION-PWY: Nitrate reduction I (denitrification), PWY490-3: Nitrate reduction VI (assimilatory), PWY-1541: Superpathway of taurine degradation, PWY-4984: Urea cycle). Correlation coefficients are shown in Supplementary Table 2

Figure 5 shows microbial genera that have high positive or negative correlation coefficients (above the threshold of 0.70, see also Supplementary Table 2) with ruminal fermentation variables or targeted pathways. Among the fermentation variables, pH (pH10) was positively associated while the concentration of total VFA was negatively associated with *Succinivibrio* and *Methanimicrococcus*. Ammonia concentration adjusted with degraded CP (i.e., NH3CP in Fig. 5) was positively associated with *Succinivibrio*. The percentage of isovalerate (isoValerate10_p) was positively associated with *Clavibacter* and FD2005 and the degradation of CP (DCP) with *Mollicutes* RF39 and *Saccharofermentans*. The latter genus was positively associated with PWY-5677 (succinate fermentation to butanoate) and PWY-5022 (4-aminobutanote degradation V). Many genera were positively associated with pathways responsible for the synthesis of acetate and butyrate. *Mogibacterium* showed a very high positive correlation (above 0.90) with P163-PWY (*L*-lysine fermentation to acetate and butanoate), PWY-5022, PWY-5676 (succinate fermentation to butanoate), PWY-5677, and, to a lower extent (coefficient 0.76–0.82), with CENTFERM-PWY (pyruvate fermentation to butanoate) and P162-PWY (*L*-glutamate degradation pathway V). Multiple genera suppressed by EXT including *Methanobrevibacter*, *Olsenella* (also decreased with GP-high), *Lachnospiraceae* NK3A20 group, *Succiniclasticum*, *Ruminococcaceae* UCG-010, and *Candidatus Saccharimonas* were correlated with pathways involved in acetate and butyrate synthesis. *Acidaminococcus*, the genus that was increased exclusively with GP treatments, was associated with PWY-190.3 (nitrate reduction VI) and P162-PWY.

## Discussion

Flavonoids including three-ring molecules and polymeric structures are the dominant groups of soluble phenolics in grapes [2]. Significant amounts of the total phenolic content remain in the grape pomace after wine production [31]. In the present work, increasing the winery by-products in the diet increased dietary phenol contents. At 20% of diet DM, the winery by-products supplied close to 3% of total grape phenols in diet DM. The most striking effect of the inclusion of grape and winery by-products was the decrease in ammonia concentration. The effect was stronger in treatments (EXT and GP-high) supplying higher phenol contents, thereby indicating a phenolic dosage effect. The ammonia-lowering effect of EXT and GP-high was accompanied by a decrease in CP disappearance. Phenolic compounds bind soluble protein under pH conditions within the ruminal range [32], therefore reducing excessive protein degradation in the rumen, which has been frequently described for tannins [33, 34]. Unlike GS and GP treatments, the effect detected with EXT ruled out the contribution of dietary shifts in physical and nutritional properties associated with the inclusion of winery by-products. Our data thus provides evidence for the role of grape bioactive compounds in the decreasing loss of dietary protein to rumen microbial fermentation. This was in line with the previous work on dairy cows describing the effects of extracts of grape pomace and grape seed on increasing the flux of protein to the small intestine to be responsible for the increased milk yield [2]. We further showed that when normalized by the unit of CP degraded, all treatments performed similarly, leading to about a 14%–18% reduction of ammonia concentration. In vitro conditions exclude the contribution of the host’s rumen absorption to the ruminal ammonia pool. Thus, our data suggest that grape phenolics, even at low dosages, can alter intermediate steps of N metabolism in the rumen such as deamination and utilization of ammonia for microbial protein synthesis. However, this assumption should be viewed with caution since the current in vitro work did not measure microbial protein synthesis. At high dosages of about 3% of diet DM (GP-high and EXT); they can decrease the degradation of dietary protein and therefore further suppress ammonia production. However, the ammonia suppression could not be associated with or explained by the microbial shifts observed in the present study. We detected strong, often negative, effects of EXT on the relative abundances at high and low taxonomic levels. The effect of GP-high, though more frequent than other GS and GP-low treatments, was seldom and weaker compared with those of EXT. Yet both EXT and GP-high showed a similar effect at a functional level related to N metabolism. Notably, studies showed inconsistent results regarding an ammonia-lowering effect of winery by-product feeding as summarized in a present review [5], which might be related to differences in dietary factors among studies. The current study revealed that winery by-products could be a dietary option when using feed sources rich in ruminally degradable protein.

Our data points out that high dosages of grape phenols can affect the ruminal fermentation of carbohydrates. Specifically, EXT and GP-high increased acetate at the expense of butyrate. In agreement, our previous study found a similar effect of grape pomace fortified in dried distillers’ grains plus solubles [7]. In the current work, all GS and GP treatments had higher contents of NDF, which promotes acetate production [35]. However, only GP-high and EXT, both providing similarly high phenol contents of around 3% of diet DM, altered the VFA profile. This hints that it was not the fiber of the winery by-products but the high phenol dosages that influenced the VFA pathways. Grape phenols could influence the VFA profile possibly via the modulation of N metabolism, considering that amino acids can be deaminated to VFA, CO_2_, and ammonia [36]. In line with that, both EXT and GP-high decreased proportions of odd and branched-chain VFA, which are products of oxidative deamination of branched-chain amino acids [37]. It must be noted that increased dietary lignin when using by-products should also be considered. Our data showed that fiber degradation was decreased when including grape pomace compared to grape seed meal. Grape pomace consists of the stems, skin, and seed of the grape berry. The presence of lignified stems in grape pomace may explain the current results.

When combining the network analysis and treatment-induced microbiota data, it revealed some players in carbohydrates and protein fermentation that can lead to acetate and butyrate shifts. Both EXT and GP-high increased *Ruminobacter,* which produces succinate, acetate, and formate [38]. On the contrary, both treatments decreased *Anaerostipes* and *Olsenella*. The latter genus was among the top ten abundant core microbiota in our samples*.* The network analysis revealed that this genus showed very high correlations with several pathways associated with butyrate production, including succinate fermentation to butyrate (PWY-5677), acetyl Co-A fermentation to butyrate (PWY-5676), *L*-lysine fermentation to acetate, butyrate and ammonia (P163-PWY) and 4-aminobutanoate degradation V producing butyrate (PWY-5022). *Olsenella* has been reported to be correlated to feed efficiency in ruminants but the findings among studies are controversial [39]. *Anaerostipes* produce mainly acetate, lactate, and butyrate from glucose [40]. It should be noted that decreased *Anaerostipe* and increased *Ruminobacter* were also observed by GP-low which did not affect the VFA composition. At the species level, the present data showed that butyrate-producing species *Butyrivibrio fibrisolvens* [41] and succinate-producing rumen spirochete *Treponema bryantii* [42] were drastically decreased by EXT. On the contrary, *Fibrobacter succinogenes* subsp. *succinogenes* increased with both EXT and GP-high. This rumen cellulolytic species produces acetate and succinate [43]. Some members of *Prevotella* also increased in EXT, suggesting that these rumen bacteria can tolerate grape phenolics and possibly outcompete some other sensitive species. Overall, EXT led to a bigger shift of ruminal bacterial composition as compared to GP-high, but both treatments showed comparable effects functionally. It seems that changes occurring to fewer members might have been sufficient to facilitate the changes in VFA production.

Different from previous findings [6, 7], we did not observe changes in methane formation with any of the treatments. The absent treatment effect on methane was in line with the VFA profiles observed, i.e., unaffected propionate production, while the effect of acetate was leveled out by butyrate. Formation of acetate and butyrate results in hydrogen that subsequently is used for methanogenesis [44]. A previous meta-analysis [8] showed that the dietary NDF content can counteract the effect of bioactive compound dosage on methane variables. While this may support our findings of GS and GP treatments, it did not explain the absent effect of EXT on methane formation. Flavonoids can influence methanogenesis via other routes such as acting as a hydrogen sink and inhibiting methanogens [1]. In our study, EXT decreased *Methanosphaera* and *Methanobrevibacter* without affecting methane formation. However, archaeal phylogeny may not be a predictor of their activity [45], and the abundance of some other players like anaerobic fungi and bacteria may be more influential [7]. The potential of grape phenols on mitigating methane may be related to the level of certain active compounds such as procyanidins and the supplementation method. For instance, adding a purified form of grape procyanidins strongly decreased methanogenesis in vitro [46]. Previously, we were able to detect a methane mitigating effect of 5% grape seed meal in the diet when fortified with dried distillers grains plus solubles [7]. It is interesting to evaluate whether the close contact of bioactive compounds with feed ingredients can stimulate an inhibitory effect on methanogenesis in the rumen.

Grape seed contains higher concentrations of polyphenols than other parts of the grapevine, except for anthocyanins, which are mainly found in the skins of red wine grapes [47]. It is conceivable that the grape pomace and grape seed meal used in our study had different phenolic profiles regardless of their similar total phenol contents and antioxidant capacity. Still, we observed consistent effects or no effect on ruminal fermentation of treatments with similar phenol contents (i.e., EXT vs. GP-high and GS-high and GP-low). This suggests that the dosage of grape phenols plays a dominant role in modulating ruminal fermentation characteristics.

## Conclusion

Our findings suggest that grape pomace and grape seed meal can be included in cattle diets, up to 20% of diet DM, without a major reduction in ruminal fermentation. In addition, grape phenols possess a functional effect on modulating ruminal N metabolism, thereby lowering ammonia production. Thus, grape and winery by-products can be recommended to be paired with feed ingredients rich in highly digestible protein. The dosage, rather than the form or source of grape phenols, is a dominant factor determining its effect in the rumen. Provision of grape phenols at 3% of diet DM is effective yet tolerable to ruminal microbiota. The recommended dosage should be proven in animal experiments taking into account the acceptance by animals as well as the epithelial response upon exposure to bioactive compounds. The present in vitro work shows the possibilities to harness the benefits of common food industry by-products which are sources of polyphenols as functional feeds for ruminants. Nevertheless, in vitro findings do not involve an influence of host-dependent factors. The results observed should be verified in future research.

## Supplementary Information

**Additional file 1:** **Supplementary Table 1.** Short-chain fatty acid concentrationof the strained ruminal fluid of each donor cows. **Supplementary Table 2.** Bacterial genera with high positive or negativewith ruminal fermentation variables or targeted pathways related to N metabolism and short-chain fatty acidsaccording to a network analysis.

## Data Availability

The data of the current study are available from the corresponding author upon reasonable request.

## Abbreviations

ADF: Acid detergent fiber
CP: Crude protein
DM: Dry matter
EE: Ether extract
FRAP: Ferric reducing antioxidant power
NDF: Neutral detergent fiber
OM: Organic matter
RUSITEC: Rumen simulation technique
VFA: Volatile fatty acid
