# Supplementary material for: Winery by-products as a feed source with functional properties: dose–response effect of grape pomace, grape seed meal, and grape seed extract on rumen microbial community and their fermentation activity in RUSITEC
Source: J Anim Sci Biotechnol. 2023 Jul 10;14:92. doi: 10.1186/s40104-023-00892-7 (PMC10332069; doi:10.1186/s40104-023-00892-7)
Supplement: Supplementary file 1 — Additional file 1: Supplementary Table 1. Short-chain fatty acid concentrationof the strained ruminal fluid of each donor cows. Supplementary Table 2. Bacterial genera with high positive or negativewith ruminal fermentation variables or targeted pathways related to N metabolism and short-chain fatty acidsaccording to a network analysis. [file 40104_2023_892_MOESM1_ESM.pdf]

Supplementary Table 1. Short-chain fatty acid concentration (mmol/L) of the strained ruminal fluid of each donor cows

Item	Acetate	Propionate	Butyrate	Isobuytrate	Valerate	Isovalerate	Caproate
Cow 1 - Run1	66.98	17.38	0.64	12.72	0.66	1.17	0.44
Cow 2 - Run1	64.33	18.92	0.70	13.45	0.78	1.33	0.51
Cow 1 - Run2	68.01	18.23	0.70	11.02	0.64	1.04	0.35
Cow 2 - Run2	62.60	18.46	0.73	9.99	0.73	0.86	0.21
Cow 1 – Run3	71.58	14.94	0.86	9.21	0.86	1.75	0.79
Cow 2 – Run3	71.52	16.19	1.04	8.68	1.03	1.22	0.32
Cow 1 - Run4	68.57	15.84	0.73	12.15	1.28	1.11	0.31
Cow 2 - Run4	67.89	17.51	0.57	11.90	0.80	1.13	0.21
Cow 1 - Average	67.86	17.15	0.69	11.97	0.86	1.11	0.37
Cow 2 - Average	64.94	18.29	0.67	11.78	0.77	1.10	0.31

Supplementary Table 2. Bacterial genera with high positive or negative (threshold of 0.70) with ruminal fermentation variables or targeted pathways related to N metabolism and short-chain fatty acids (SCFA) according to a network analysis

Pathway or ruminal fermentation vairable*	Bacterial genera	Correlation coefficient
CENTFERM-PWY	Enterorhabdus	0.731
	Rikenellaceae_RC9_gut_group	0.916
	Christensenellaceae_R.7_group	0.702
	Mogibac	0.816
	Lachnospiraceae_UCG.008	0.799
	Ruminococcaceae_UCG.002	0.862
	Ruminococcaceae_UCG.010	0.782
	Succiniclasticum	0.828
PWY490-3	Rikenellaceae_RC9_gut_group	0.785
	Ruminococcaceae_UCG.002	0.773
	Ruminococcaceae_UCG.010	0.753
	Acidaminococcus	0.843
P163-PWY	Methanobrevibacter	0.809
	Atopobium	0.801
	Olsenella	0.791
	Enterorhabdus	0.759
	Rikenellaceae_RC9_gut_group	0.841
	Christensenellaceae_R.7_group	0.833
	Mogibac	0.955
	X.Eubac._hallii_group	0.764
	Lachnospiraceae_NK3A20_group	0.727
	Lachnospiraceae_UCG.008	0.815
	probable_genus_10	0.739
	Syntrophococcus	0.765
	Ruminococcaceae_UCG.002	0.828
	Ruminococcaceae_UCG.010	0.865
	X.Anaerorhabdus._furcosa_group	0.732
	Succiniclasticum	0.907
	Candidatus_Saccharimonas	0.816
	p.1088.a5_gut_group	0.791
P162-PWY	Rikenellaceae_RC9_gut_group	0.768
	Mogibac	0.762
	X.Eubac._hallii_group	0.708
	Ruminiclostridium_1	0.719
	Ruminococcaceae_UCG.002	0.765
	Ruminococcaceae_UCG.010	0.827
	Acidaminococcus	0.883
	Succiniclasticum	0.756
PWY-5022	Methanobrevibacter	0.760
	Olsenella	0.887
	Christensenellaceae_R.7_group	0.751
	Mogibac	0.937
	Lachnospiraceae_NK3A20_group	0.805

	Lachnospiraceae_NK4A136_group	0.726
	probable_genus_10	0.721
	Syntrophococcus	0.828
	Ruminococcaceae_UCG.010	0.788
	Succiniclasticum	0.850
	Candidatus_Saccharimonas	0.793
	p.1088.a5_gut_group	0.728
PWY-5677	Methanobrevibacter	0.726
	Olsenella	0.825
	Mogibac	0.910
	X.Eubac._ventriosum_group	0.729
	Lachnospiraceae_NK3A20_group	0.756
	Syntrophococcus	0.780
	Ruminococcaceae_UCG.010	0.723
	Saccharofermentans	0.719
	Succiniclasticum	0.786
	Candidatus_Saccharimonas	0.791
PWY-5676	Methanobrevibacter	0.793
	Olsenella	0.803
	Enterorhabdus	0.718
	Mogibac	0.913
	Lachnospiraceae_NK3A20_group	0.800
	probable_genus_10	0.789
	Syntrophococcus	0.715
	Succiniclasticum	0.919
	Candidatus_Saccharimonas	0.704
pH	Methanimicrococcus	0.740
	Succinivibrio	0.713
	WPS.2_unc_ru_bac_unc_ru_bac_ unc_ru_bac_unc_ru_bac	0.743
SCFA concentration	Methanimicrococcus	-0.817
	Bacteroidales_bac_Bact_22	-0.738
	Succinivibrio	-0.737
Iso-valerate (molar %)	Clavibacter	0.736
	FD2005	0.795
CP degradation (% of supply)	Saccharofermentans	0.709
	Mollicutes_RF39_unc_ru_bac_unc_ru_bac	0.714
NH3 (mmol/g degraded CP)	Succinivibrio	0.717

*CENTFERM-PWY: pyruvate fermentation to butanoate, P162-PWY: L-glutamate degradation V (via hydroxyglutarate), P163-PWY: L-lysine fermentation to acetate and butanoate, PWY-5022: 4-aminobutanoate degradation V, PWY-5676: Acetyl-CoA fermentation to butanoate II, PWY-5677: Succinate fermentation to butanoate, DENITRIFICATION-PWY: Nitrate reduction I (denitrification), PWY490-3: Nitrate reduction VI (assimilatory), PWY-1541: Superpathway of taurine degradation, PWY-4984: Urea cycle
